# Olfactory markers using ScentAware in LMICs

**DOI:** 10.1002/alz70860_097263

**Published:** 2025-12-23

**Authors:** Subhanjan Mondal

**Affiliations:** ^1^ SensifyLife, Middleton, WI, USA

## Abstract

The connection between Alzheimer's disease (AD) and olfactory deficits is well documented. Olfactory identification deficits are linked to tau accumulation in key areas of the brain associated with olfaction, with strong predictive power for longitudinal tau progression. Pen‐and‐paper versions of olfactory identification have been used in research on neurodegenerative diseases. Considering the advantages of digital platforms in simplifying workflow, longitudinal data capture and allowing self‐assessment, we developed ScentAware, a digital olfactory assessment system. The system comprises three components: 1) the ScentAware olfactory test kit 2) SensifyAware mobile app, and 3) SensifyAware cloud‐based data management and analytics platform. In the ScentAware olfactory test kit, olfactory stimulants are contained within tubes that are labelled with a QR‐code. The 16 olfactory stimulants in the ScentAware kits are orange, lemon, rose, lavender, clove, cinnamon, garlic, banana, peppermint, smoke, wood, coffee, leather, peppermint, eucalyptus, pineapple. Scanning of the QR‐code using the SensifyAware app using a mobile device initiates a forced‐choice questionnaire. Participants identify odors from four options, or an option for no odor detected. Responses are captured digitally and retrieved from the cloud‐data management system for analysis.

The ScentAware platform is being validated in three DAC cohorts in Kenya, Egypt and India representing a diversity of socioeconomic classes. One of the approaches we took to address the issue of cultural adaptation was to create the test in local languages, Swahili for Kenya and Arabic for the Egypt cohort. We also approached cultural adaptation by having options that are more familiar to the populations. For the study in India, we are using an abbreviated version of the test using eight items that were found to be easily identified by the local population in a pilot study. User engagement and acceptance in doing the ScentAware test was one feature that stood out from the data collection efforts. The completion of the test was high for cognitively normal groups. The test showed acceptable internal consistency (Cronbach's α =0.711). All three studies are ongoing, results from interim analysis from some of the cohorts will be presented at the conference.

